# Ultrastructural Changes of Neuroendocrine Pheochromocytoma Cell Line PC-12 Exposed In Vitro to Rotenone

**DOI:** 10.3390/brainsci14050476

**Published:** 2024-05-08

**Authors:** Manuel Belli, Mario Cristina, Valeria Calabrese, Marta Russo, Marisa Granato, Matteo Antonio Russo, Luigi Sansone

**Affiliations:** 1 Department of Human Sciences and Promotion of the Quality of Life, San Raffaele Roma Open University, 00166 Rome, Italy; manuel.belli@uniroma5.it (M.B.); granatomarisa@gmail.com (M.G.); 2 Laboratory of Molecular, Cellular and Ultrastructural Pathology, IRCCS San Raffaele Roma, 00166 Rome, Italy; mario.cristina@uniroma1.it (M.C.); matteoantoniorusso44@gmail.com (M.A.R.); 3 Department of Molecular Medicine, University La Sapienza, Viale del Policlinico 155, 00161 Rome, Italy; 4 Experimental Neurophysiology Laboratory, IRCCS San Raffaele Roma, 00166 Rome, Italy; valeria.calabrese@sanraffaele.it (V.C.); marta.russo@sanraffaele.it (M.R.)

**Keywords:** PC12, rotenone, transmission electron microscopy, neurotoxicity, neuropeptide granules, synaptic vesicle, cellular and molecular rehabilitation

## Abstract

Rotenone is a pesticide used in research for its ability to induce changes similar, in vivo and in vitro, to those observed in Parkinson’s disease (PD). This includes a selective death of dopaminergic neurons in the substantia nigra. Nonetheless, the precise mechanism through which rotenone modifies structure and function of neurons remains unclear. The PC12 cells closely resemble dopamine terminal neurons. This makes it a preferred model for studying the morphology of central dopamine neurons and predicting neurotoxicity. In this paper, we investigated the effects of 0.5 µM rotenone for 24–48 h on PC12 cell viability and ultrastructure (TEM), trying to identify primary and more evident alterations that can be related to neuronal damages similar to that seen in animal PD models. Cell viability decreased after 24 h rotenone treatment, with a further decrease after 48 h. Ultrastructural changes included vacuolar degeneration, mitochondrial mild swelling, decrease in the number of neuropeptide granules, and the loss of cell-to-cell adhesion. These findings are in agreement with previous research suggesting that rotenone, by inhibiting energy production and increasing ROS generation, is responsible for significant alterations of the ultrastructure and cell death of PC12 cells. Our data confirm the link between rotenone exposure, neuronal damage, and changes in dopamine metabolism, suggesting its role in the pathogenesis of PD.

## 1. Introduction

Although the etiology of Parkinson’s disease (PD) is not completely understood, it is accepted that in its pathogenesis, an important role is played by interactions between genetic and environmental factors [1]. Exposure to certain pesticides found in the environment has been linked to an elevated risk of PD, likely due to their mitochondrial toxicity, as indicated by epidemiological research [2,3,4]. Rotenone ranks among the extensively utilized pesticides globally. Classified by the World Health Organization as a moderately hazardous substance (a class II pesticide), rotenone rarely causes acute poisoning in humans. This rarity stems from its notably higher estimated oral LD50 in humans (300–500 mg/kg b.w.) compared to the chronic exposure experienced environmentally [5]. However, epidemiological evidence also suggests that rotenone chronic exposure represents a risk factor for PD pathogenesis [6]. Betarbet et al. [7] successfully replicated many of the clinical, biochemical, and pathological characteristics associated with PD in rats chronically exposed to rotenone. Since then, rotenone has garnered significant interest due to its potential role as an environmental neurotoxin involved in the development of PD. Furthermore, it has become a widely used experimental model for studying the basic mechanisms contributing to neuronal damage leading to PD and assessing potential novel treatments for this condition [8].

For its remarkable lipophilicity, rotenone easily traverses biological barriers, such as blood–brain barrier, and cell membranes, including the outer and inner mitochondrial membranes, lysosomes, endoplasmic reticulum (ER), and Golgi [9]. It produces its major toxic effects at the level of mitochondrial cristae by specifically blocking complex I of the respiratory chain and decreasing mitochondrial adenosine triphosphate (ATP) production by 30% [10]. As a consequent effect, it increases the mitochondrial production of reactive oxygen species (ROS) which can rapidly diffuse to the other subcellular compartments through voltage-dependent anion channel (VDAC) and mitochondrial aquaporins [11]. The decrease of energy charge and the detrimental effects of ROS are responsible for a number of PD-related subcellular neuronal changes in the substantia nigra. In particular, mitochondrial swelling and opening of mitochondrial VDAC, release of cytochrome C and APAF, activating apoptosis and other so-called “dopaminergic cell death”; oxidative stress produces α-synuclein phosphorylation and aggregation, protein deglycase (DJ-1) acidification and translocation, dysfunction of the proteasome system, and the accumulation of iron in the substantia nigra [12].

At a clinical level, it replicates certain non-motor symptoms of PD, particularly disruptions in gastrointestinal function and olfactory discrimination [13]. Its utility in evaluating various endpoints such as α-synuclein accumulation, and the functioning of the ubiquitin–proteasome system, in addition to its capacity to preserve dopaminergic neurons and related motor functions. These are among the most commonly utilized endpoints in studies focused on neuroprotection [14]. These effects of rotenone can be traced back to 1968 when Palmer and collaborators discovered that the primary effect consisted of hindering the transfer of electrons from the iron–sulfur midpoints in complex I to ubiquinone. This inhibition resulted in the disruption of electron transfer along the respiratory chain with a decreased production of ATP [8].

More recently, Peng and collaborators [15] reported that exposure to rotenone induces changes in mitochondrial biogenesis and dynamics by reducing the cellular levels of peroxisome proliferator-activated receptor gamma coactivator 1-alpha (PGC1α), which controls mitochondrial growth, fission, and fusion. 

In another study, it was demonstrated that inhibiting fission effectively decreased rotenone-induced neurotoxicity in primary neurons [15]. Rotenone, following mitochondrial damage, can trigger apoptosis and other types of cell death through diverse signaling pathways, including the AKT/Glycogen synthase kinase-3β (GSK-3β) pathway [16]. Cofilin, a crucial modulator of actin filament dynamics, and tubulin have been observed after mitochondrial damage [17]. Specifically, de-phosphorylated cofilin, tubulin, and some tubulin-associated proteins relocate from the cytoplasm to the mitochondria before the release of cytochrome c, ultimately aggravating mitochondrial damage [18,19].

The primary objective of our research is to investigate the PC12 cell line, as model of dopamine neurons, the acute (24–48 h) toxic effects of low-dose of rotenone [20,21] on viability and ultrastructural changes which appears the most evident association between environmental exposure and the degeneration of dopaminergic neurons [22]. Further insight detailing the risk of rotenone exposure to the broader population and of the mechanism(s) of damage will be useful to define this cell line as a model system to explore the physiology of central dopamine neurons and to assess the potential neurotoxicity of other compounds that affect these neurons and identify precise and personalized therapeutic targets [23,24].

## 2. Materials and Methods

### 2.1. Cell Culture

PC-12 (CCL-185 ATCC) cells were purchased by ATCC and grown as monolayers following the ATCC’s protocols. Cells were grown in DMEM/F12 medium (Gibco, Thermo Fisher Scientific, Waltham, MA, USA) at 37 °C in a 5% CO_2_. The culture medium was supplemented with 5% heat-inactivated fetal bovine serum, 100 IU/mL penicillin, and 100 µg/mL streptomycin (Gibco, Thermo Fisher Scientific, Waltham, MA, USA). Cells were seeded in 25 cm^2^ flasks at a density of 5 × 104 cells/flask. The media was refreshed every 2–3 days. The experiments were performed on the specimens between passage 15 and 25. The treatments with/without rotenone (0.5 µM; Sigma-Alderich, St. Louis, MO, USA) for 24 h or 48 h were accomplished 3–8 days after subculture. Rotenone was dissolved in dimethyl sulfoxide (DMSO, Sigma-Alderich, St. Louis, MO, USA). Following the removal of the supernatant from PC12, they were rinsed with PBS, followed by treatment with Trypsin-EDTA (Gibco, Thermo Fisher Scientific, Waltham, MA, USA), and then incubated for 1 min at 37 °C. Subsequently, the cells were transferred to a centrifuge tube, where complete medium was added to neutralize the trypsin-EDTA, and the cells were centrifuged for 5 min at 1200 RPM. The supernatant was removed, cells were resuspended in PBS, and were centrifuged for 5 min at 1200 RPM. The supernatant was removed, the cells were resuspended in a glutaraldehyde solution, and stored at +4 °C.

### 2.2. Cell Viability

Cells were initially seeded in a 100 mm dish and treated according to the specified protocol upon reaching 80–90% confluence. Afterward, at 24 and 48 h time points, cells were harvested and combined with Trypan blue at a dilution ratio of 1:2. The resultant cell suspension was then examined using a hemocytometer under phase contrast microscopy for quantification. The experiments were conducted twice, with each condition analyzed in triplicate. The number of cells was calculated using the formula N° cells × mL = (N° cells/N° quadrants) × 200 × 1000 following cell counting using a Burker chamber. For each condition in triplicate, both live and dead cells were counted and compared.

### 2.3. Transmission Electron Microscopy

PC12, after diverse treatments, were collected and fixed overnight in 2.5% glutaraldehyde with 0.1 M sodium hydroxide, 0.1 M, pH 7.3 [25]. Samples were washed 6 times in the sodium hydroxide buffer, and then post-fixed in 2% osmium tetroxide in the same buffer for 2 h at room temperature and treated following a standard protocol for embedding in EPON resin [26]. Next, polymerization procedure overnight at 65 °C was performed, ultrathin sections of 80 nm of thickness, were cut on a Leica Ultracut E Ultramicrotome (Leica Microsystems, Wetzlar, Germany) and placed on copper grids, contrasted with UranyLess stain and lead hydroxide, and, lastly, examined in a JEOL-1400 Plus TEM (Jeol Ltd., Tokyo, Japan).

### 2.4. Morphometric Analysis

Mitochondria and neuropeptide granule numerical density was evaluated as previously reported [27]. Briefly, at least 15 specimens per group were imaged using TEM at the same magnification (2500×) on at least five equatorial sections (distance between the sections: 3–4 μm) and analyzed by means of ImageJ 1.53 software (http://rsbweb.nih.gov/ij/, accessed on 20 January 2023). The digital images were additionally magnified to facilitate the identification and enumeration of the organelles. Values are expressed as numerical density per cell or linear surface. Mitochondrial length was measured as previously reported [28].

### 2.5. Statistical Analysis

The data are presented as means ± standard deviation (SD). Statistical comparisons were conducted utilizing either one-way or two-way ANOVA, followed by Tukey’s HSD tests for post hoc evaluation (GraphPad InStat or Prism 5, La Jolla, CA, USA). Significance was determined at *p* < 0.05. Variance between groups was assessed utilizing the Levene test.

## 3. Results

### 3.1. Effect on Viability 

Viability tests were performed in duplicate. The results of the Trypan blue test indicated elevated mortality in the samples treated with rotenone compared to the controls. Additionally, mortality appeared to be time-dependent, with a higher incidence observed in the 48 h condition compared to the 24 h condition (Table 1).

### 3.2. Ultrastructural Evaluation

#### 3.2.1. PC12 Control

Using light microscopy (LM), control cells appeared small (10/µm diameter), spherical, and arranged in adjacent clusters. These cells showed a high nucleus/cytoplasm ratio. Nuclei, surrounded by a prominently stained nuclear membrane, showed one or more nucleoli. Additionally, certain cells displayed short and pointed extensions (Supplementary Figure S1).

**Figure 1:**
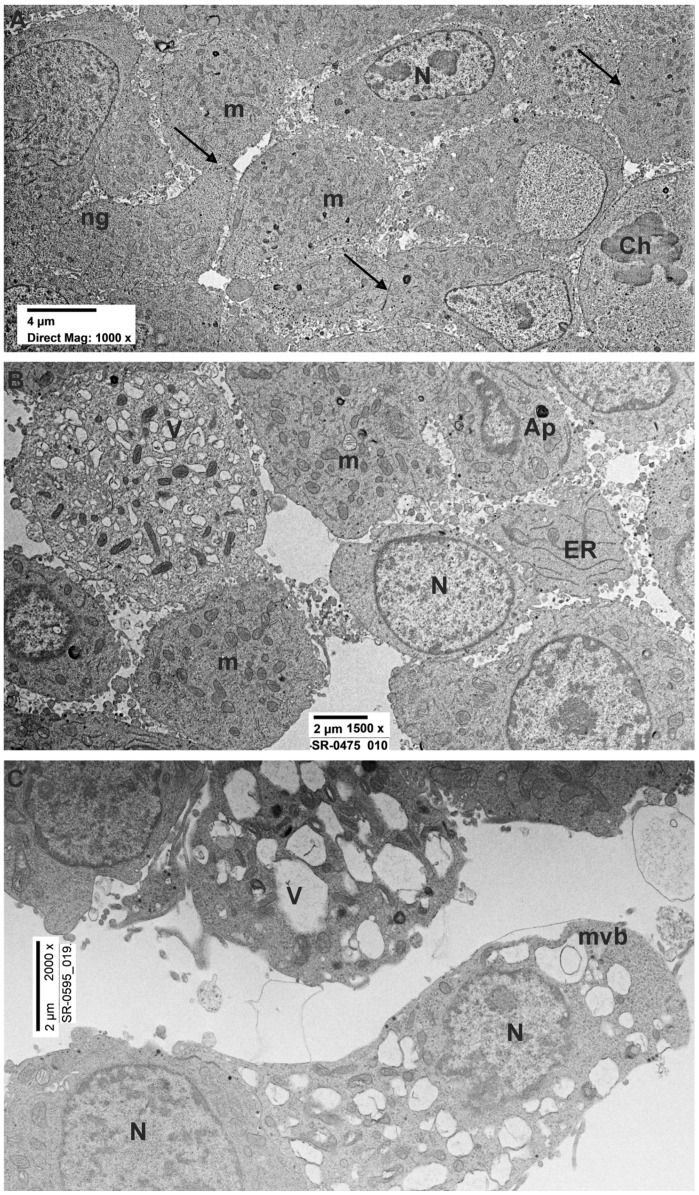
Ultrastructural evaluation of untreated, rotenone 24–48 h PC12 cells. (**A**) Ultrastructure of control PC12s showing evident nuclei (N) delimited by a continuous nuclear membrane and numerous round/ovoid mitochondria (m). Intercellular contacts (arrows) were present among the cells. Numerous neuropeptides granules (ng) were visible. (**B**) Ultrastructure of PC12 treated with rotenone 0.5 µM for 24 h showing large nuclei delimited by an intact nuclear membrane and numerous mitochondria. Intercellular contacts among the cells were partially lost. Degenerating cells with several vacuoles (V) were present. (**C**) Representative TEM micrograph of PC12 treated with rotenone 0.5 µM for 48 h displaying clear signs of cell degeneration. Numerous vacuoles and isolated multivesicular bodies (mvb) were visible. Ch: chromosomes; ap: autophagosome; ER: endoplasmic reticulum.

**Figure 2:**
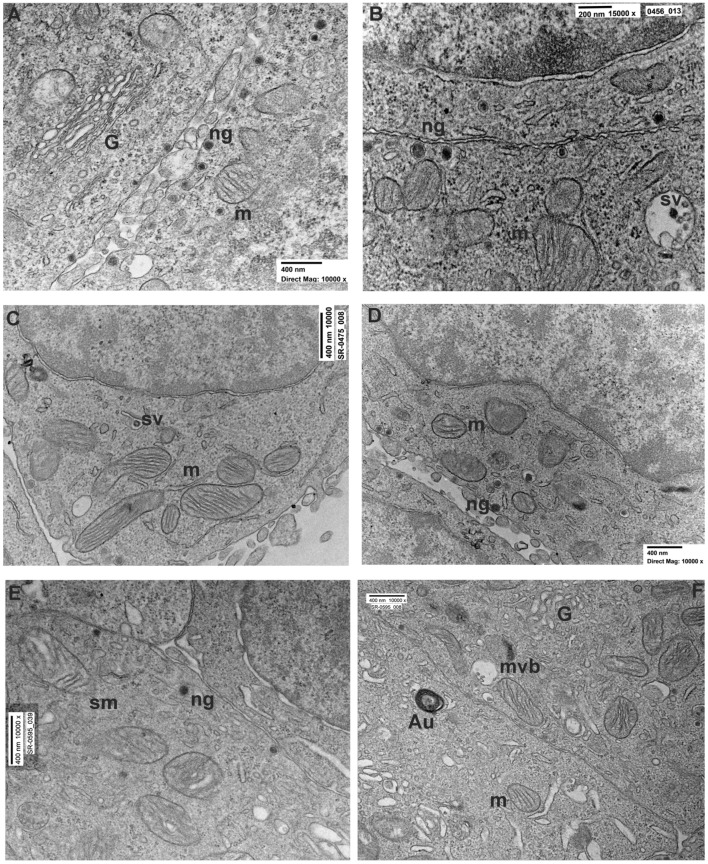
Ultrastructural comparison of neuropeptide granules and synaptic vesicles in untreated and rotenone 24–48 h PC12 cells. (**A**,**B**) High magnification of two cell borders in the untreated PC12 group. Note the presence of numerous neuropeptides granules (ng) attached to the plasma membrane. Numerous Golgi cisternae (G) and mitochondria (m) with visible cristae were observed. (**C**,**D**) Representative picture of PC12s treated with rotenone 0.5 µM for 24 h showing the reduced presence of neuropeptide granules in proximity of the cell borders and elongated mitochondria with evident cristae. (**E**,**F**) Ultrastructure of PC12 treated with rotenone 0.5 µM for 48 h showing an evident reduction of neuropeptide granules, mitochondria with sign of swelling (sm), and multivesicular bodies (mvb) containing altered synaptic vesicle (sv).

Using transmission electron microscopy (TEM), control cells displayed a distinct arrangement resembling epithelial cells. (Figure 1A). The surfaces of adjacent cells were in close proximity (15–30 nm) and occasionally connected by adhering regions. Beneath the plasmalemma, there was a sparse cortex of amorphous material where occasional microfilaments could be identified. Several cells showed the presence of numerous long microvilli on the external surface. The nuclei appeared eccentric with patches of heterochromatin (Figure 1A). Microtubules and profiles of endoplasmic reticulum (ER) were occasionally seen. The cytoplasm of the cells showed numerous neuropeptidic dense granules, synaptic vesicles (Figure 2A,B), mitochondria (Figure 2A,B), and ribosomes. Occasionally, the TEM micrograph showed lysosomes with a moderately dense matrix, sometimes containing a myelin-like structure. Numerous Golgi cisternae were detected (Figure 2A).

#### 3.2.2. PC12 Treated with Rotenone 0.5 µM for 24 h 

By LM, treated PC12 cells appeared small with a spherical shape. They rarely appeared organized in clusters. Similarly to the previous group, these cells presented a high nucleus/cytoplasm ratio. Nuclei, bordered by an intact nuclear membrane, exhibited one or more nucleoli (Supplementary Figure S1). 

Cells treated with rotenone 0.5 µM for 24 h showed sublethal ultrastructural changes with respect to the untreated cells. The major change was observed in moderately swollen cells (20–25%) with dilated ER cisternae, condensed or swollen mitochondria, vacuoles, and a decreased number of both dense granules and synaptic vesicles both in the cytosol and at the secretory border. However, most of PC12 cells showed a round-to-ovoid shape with evident roundish nuclei (Figure 1B). Chromatin distribution did not change from that of the controls, with spots of heterochromatin usually placed in the middle of the cell or, more infrequently, were eccentric. The cytoplasm exhibited the presence of several round/ovoid mitochondria bordered by a double electron-dense mitochondrial membrane with numerous platform mitochondrial cristae (Figure 2C,D). The observation also revealed the reduction of intercellular connections through junctions and projections with respect to the control group (Figure 1B). Furthermore, the cytoplasm of these cells presented microfilaments, occasional microtubules and intermediate filaments, and tubules of ER. 

Electron micrographs showed also the presence of multivesicular bodies, vesicles, autophagosomes, and lysosomes (Figure 2C,D). Small granules and synaptic-like vesicles, which were extremely common in untreated PC12 cells, were minorly present in this experimental group (Figure 2C,D).

#### 3.2.3. PC12 Treated with Rotenone 0.5 µM for 48 h

By LM, after 48 h rotenone treatment, PC12 cells exhibited a spherical morphology and reduced appearance with infrequent clustering and notable large intercellular gaps. An increased high nucleus/cytoplasm ratio was observed (Supplementary Figure S1).

By TEM, cells treated with rotenone for 48 h showed more evident ultrastructural changes as compared to the control and to the 24 h rotenone treatment groups. In TEM micrographs (Figure 1C) evaluation (Table 2), 48 h-treated cells showed the presence of degenerating (swollen) cells (30–35%) with a number of subcellular changes. In particular, electron-clear cytosol, fragmented ER with enlarged cisternae and detached ribosomes, decreased or swollen Golgi apparatus, and substantially decreased dense granules and synaptic vesicles/multivesicular bodies were frequent in the affected cells. Dense granules were mostly interspersed in the diluted cytosol, being only occasionally observed close to the secretory pole of the plasma membrane (Figure 2E,F). Cells only occasionally presented as multicellular clusters with close reciprocal contacts, while the extracellular space appeared definitively increased. Perinuclear Golgi apparatus, often associated with ER and vesicles, as well as with the multivesicular bodies, were seen. Similar to the rotenone 24 h group, a reduction in the presence of intercellular connections was noted (Figure 1C). Notably, mitochondrial cristae appeared less electron-dense (Figure 1E). Occasionally autophagosomes were observed, especially in swollen cells (Figure 1C and Figure 2F). Morphometry confirmed a significant decrease in the numerical density of dense granules, multivesicular bodies, and synaptic-like vesicles (Figure 2E,F). 

#### 3.2.4. Morphometric Analysis

The morphometric analysis revealed a slight downward trend in the mitochondrial numerical density from the control to the rotenone 24 and 48 h groups (C: 24.8 ± 5.4; R 24: 22.7 ± 5.8; R 48: 20 ± 5.1). The mitochondrial length evaluation did not show significant changes among the three groups (C: 0.418 ± 0.183; R24: 0.484 ± 0.173; R48: 0.478 ± 0.161). Importantly, the morphometric evaluation displayed a significant downward trend in the neuropeptide granules numerical density from control to the rotenone 24 and 48 h groups (C: 44.3 ± 16.5; R 24: 15.7 ± 11.4; R 48: 9.3 ± 14.3). We observed a similar downtrend with the evaluation of the numerical density of the granules localized in the proximity of the cell borders (C: 11.1 ± 3.2; R 24: 5.2 ± 2.4; R 48: 3.1 ± 1.7). 

## 4. Discussion

This paper aimed to present a detailed analysis of the ultrastructure and viability of PC12 exposed to rotenone in vitro. Although the control normal ultrastructural characteristics of PC12 have already been reported [29], this is the first time that PC12 cells cultured with rotenone were evaluated at the ultrastructural level by TEM and morphometry and discussed in relation to PD pathogenesis. 

Overall, we found that in the great number of cells, both the control and rotenone-treated cells, did not show major alterations in shape, size, and general organization of the cytoplasm and minor changes in subcellular organelles. However, 20–35% of rotenone-treated cells displayed general and ultrastructural changes that were more evident with longer time of treatment and strictly associated with increased cell death and with known toxic mechanisms and targets of rotenone [30,31,32].

Based on our results and on what is already published, we aim to discuss two specific points about the validity of rotenone-intoxicated PC12 cells as a cellular model for human PD: 1- What are some of potential mechanism(s) by which rotenone damage PC2 cells? 2- Comparison between the morphological characteristics that were actually analyzed; dopaminergic cells of substantia nigra seem to be a selective target of rotenone: is it true? Can other cells, such as microglia, contribute to the neuronal damage by their specific damage or activation?

Rotenone acts as a specific mitochondrial toxin, inhibiting mitochondrial electron transfer at Site 1 of the respiratory chain (complex I-NADH-dehydrogenase), with two main primary effects; a decrease in mitochondrial respiration with a reduction in ATP production and deviation of electrons from cytochrome chain to the utilization of mitochondrial oxygen with increased ROS production. Both energy charge decrease and ROS overproduction affect a number of cell structures/functions, possibly leading to cell death [33].

### 4.1. Mitochondrial Disfunction 

Following rotenone treatment, the mitochondrial matrix can be more electron-clear (moderate swelling) with plate-like cristae or more electron-dense (condensed form) with swollen irregular cristae. These modifications of the conformation have been described by Hackencbrock et al. [34] in relation to the changes of mitochondrial energy charge. In addition, morphometric data showed a decrease in the numerical density of mitochondria and an increase in mitochondrial length in long-term rotenone-treated cells as compared to the control. This finding is supported by Peng et al. [14] which demonstrated that rotenone exposure alters both mitochondrial dynamics and biogenesis. In their in vitro experiments, rotenone downregulated the cellular level of peroxisome proliferator-activated receptor-γ-coactivator 1-α, a transcription factor controlling mitochondrial biogenesis and fission/fusion processes. Furthermore, another study Arnold et al. [35] showed that rotenone alters mitochondrial morphology by first inducing fusion as an initial compensatory response, followed by damage-inducing fission. 

### 4.2. Effects of ROS and Oxidations of Other Biological Targets—Metabolic Effects

Energy production is further aggravated by the oxidation of other enzymes of glucose and pyruvate metabolism. Chiaradia et al. [36] confirmed the correlation between rotenone exposure and carbonylation of glycogen phosphorylase, pyruvate carboxylase, pyruvate kinase, transketolase, and alpha-enolase, all involved in glycolysis, pentose phosphate, and tricarboxylic acid cycle pathways. As further evidence, the oxidation of these enzymes has been reported in PD whole brain tissues [37]. Taken together, all the alterations of the energy metabolism from glycolysis to ATP synthesis may represent a relevant factor for the damage of function and maintenance of neurons, possibly linked to the occurrence of neurodegenerative disorders [38,39]. 

### 4.3. Possible Impact on Granule/Vesicle/Multivesicular Bodies Formation, Transport and Secretion, and Autophagocytosis 

Our results showed a decrease in the distribution and number/cell of both neuropeptidic dense granules and synaptic vesicles/multivesicular bodies, depending on the duration of rotenone treatment. PC12 exposed for 48 h showed a dramatic reduction in these structures (Table 3) in absolute number/cell and in their distribution into the cytosol and at the secretory pole of the cell. This may suggest both a reduction in their formation (as indicated by the decrease of Golgi complex changes) and an inhibition of their transport to the secretory pole. Formation, transport, and secretion of vesicles and granules are strongly energy-dependent and are inhibited when the energy charge falls below 60–70% or normal levels. Interestingly, recent studies show that PD-linked neurotoxins, like rotenone, determine carbonylation of specific chaperones, producing oxidative changes which affect the protein function. One of these targets is the vacuolar-type proton ATPase subunit B, brain isoform (V-ATPase), which has a role in vesicle/granule formation [40]. Other carbonylation targets are the proteasome protein subunit alpha, and transitional endoplasmic reticulum ATPase [36], and cytoskeletal elements (microtubules and actin filaments) which are crucial for vesicle formation, transport and secretion, as well as autophagy–lysosome and ubiquitin–proteasome pathways aimed for removing damaged cellular components. Prior studies have linked dysfunction in these pathways to PD pathogenesis [41,42,43].

### 4.4. Cell-to-Cell Contacts in Confluent Cultures

Our study showed a substantial decrease in cell-to-cell contact in the rotenone-exposed cells as compared to the untreated cells. A previous work supports our observations, identifying specific proteins in PC12 cells that are most vulnerable to the oxidative stress induced by rotenone and can be involved in contacts. A 2D gel electrophoresis followed by proteomic procedures and STRING characterization identified 17 oxidized proteins, implicated in different cellular processes that were affected by protein oxidative modification. One of the involved proteins was the programmed cell death 6 interacting protein (PDCD6IP) involved in membrane repair, cytokinesis, and formation of tight junction [36].

### 4.5. In Vivo/In Vitro Mechanisms and Limitations of This Paper 

**Figure 3:**
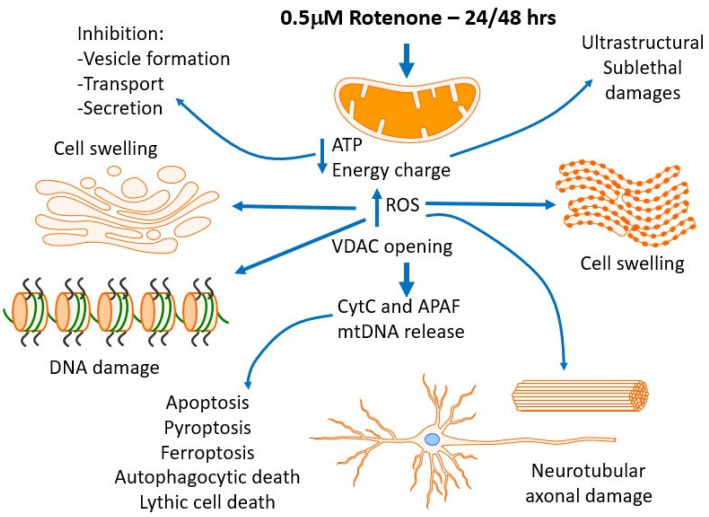
Schematic representation of rotenone’s direct and indirect mechanisms of action at different subcellular levels. Direct effects of rotenone are considered as the ATP production decrease and the increase in ROS generation. Indirect effects stem from those direct. A decrease in energy charge affects many aspects of structure/function maintenance such as cell and organellar volume control (cell swelling), vesicle formation, transport and secretion, and cytoskeletal functions. Increased ROS generation affects mitochondrial components (such membranes, VDAC, enzymes, and proteins), and then, after diffusing into the cytosol through aquaporins, may target a large number of elements, among them the Golgi and endoplasmic reticulum (contributing to ER-stress), lysosomes and peroxisomes, and cytoskeletal elements (microtubule and actin filament fragmentation) leading to cell movement and architecture changes. A combined indirect catastrophic effect is the cell death through different pathways leading to apoptosis (VDAC opening with release of cytC, APAF, mtDNA), pyroptosis [45], ferroptosis [46], and lithic/autophagocytic death (with increased autophagocytosis) [47].

With rotenone being a highly lipophilic molecule, it can cross biological membranes, such blood–brain barrier (BBB), other barriers, and cell membranes such the outer/inner mitochondrial membranes [9]. When crossing the BBB, rotenone encounters and intoxicates different cell types, including endothelial cells, fibroblasts, astrocytes, microglia, and neurons. A severe limitation of this PC12 cell line model is that in our analysis, we completely missed the interactions among different cells present in the microenvironment of the PD target neurons (substantia nigra). Recently, the role of microglial activation and damage, the activation of the inflammasome, and the different types of cell death occurring in target neurons is emerging from experiments in animals where damaging tissue signals and pathways may be explored [44] (Figure 3). Another limitation of this paper is that several of our data (presence of cellular junction, multivesicular bodies, and synaptic vesicle content) were qualitatively described and not quantitatively evaluated. Future work should also exactly quantify the relative abundance of each element. 

## 5. Conclusions

The results of our study suggest that the rotenone-treated PC12 model of neurotoxicity could be regarded as one of the fairly reproducible and simple experimental toxin-based PD models. However, considering the results of this study and the above referred limitations, further analysis is necessary to enhance our understanding the rotenone neurotoxicity when referred as PD models. Our results indicate that the toxicity of rotenone, especially after 48 h of exposure results in important morphological and phenotypic alterations that could serve as a model system for studying some aspects Parkinson’s disease.

PC12 cells can be induced to differentiate into a more neuron-like phenotype using various methods, such as exposure to nerve growth factors [48]. This differentiation process induces changes in cell morphology and functionality that renders PC12 more similar to the dopaminergic in vivo neurons. It would be interesting to demonstrate whether the differentiation of PC12 cells influences their sensitivity to rotenone. A further improvement of the PC12 cell model could be the culture of organoids and or co-culture with microglia and astroglia that could overcame the absence of inflammatory cells that recently have acquired an important role in the PD neuronal damage and in disease progression.

## Figures and Tables

**Table 1:** Viability data expressed as mean ± standard deviation.

Treatment	Cell Death (%)
Control 24 h	7.23 ± 0.95
Rotenone 24 h	14.70 ± 1.85
Control 48 h	4.65 ± 0.92
Rotenone 48 h	16.71 ± 0.40

**Table 2:** Summary of the qualitative data obtained by TEM analysis on the main ultrastructural features in PC12 unexposed (controls) or exposed to 0.5 µM of rotenone for 24 or 48 h.

		*Rotenone*	
	*Control*	*0.5 µM for 24 h*	*0.5 µM for 48 h*
**Mitochondria**	Round/ovoid shape with electron-dense cristae. Uniformly distributed.	Round/elongated shape with electron-dense cristae. Uniformly distributed.	Round/elongated shape with less electron-dense cristae. Uniformly distributed.
**Nuclei**	Roundish, delimited by an intact electron-dense nuclear membrane.	Roundish, delimited by an intact electron-dense nuclear membrane.	Roundish or irregular, delimited by an intact electron-dense nuclear membrane. Presence of nuclear invagination.
**Nuclear content of** **Hetero** **Chromatin**	Regularly distributed in the nucleus with minor condensation close the nuclear envelope.	Regularly distributed in the nucleus with minor condensation close the nuclear envelope.	Regularly distributed in the nucleus with minor condensation close the nuclear envelope.
**ER**	Usually found in form of isolated cisternae.	Usually found in form of isolated cisternae.	Usually found in form of isolated cisternae
**Neuropeptide granules**	Numerous. Mainly distributed in peripheral area of the cell.	Less numerous. Mainly distributed in peripheral area of the cell.	Rare. Mainly distributed in peripheral area of the cell.
**Intercellular connection**	Numerous.	Less numerous.	Less numerous.
**Autophagosomes and vacuoles**	Rare.	Occasionally present.	Present.

**Table 3:** Morphometric assessment (expressed as mean ± standard deviation) of organelles in control and rotenone-exposed groups. The analysis was conducted employing a one-way ANOVA with Tukey HSD post hoc analysis. Same superscripts letters indicate a significant difference (*p* < 0.05).

		*Rotenone*	
	*Control*	*0.5 µM for 24 h*	*0.5 µM for 48 h*
**Numerical density of mitochondria (per cell)**	24.8 ± 5.4 ^a^	22.7 ± 5.8 ^b^	20 ± 5.1 ^a^
**Mitochondrial length (µm)**	0.418 ± 0.183 ^a^	0.484 ± 0.173 ^b^	0.478 ± 0.161 ^c^
**Numerical density of neuropeptide granules (per cell)**	44.3 ± 16.5 ^a,b^	15.7 ± 11.4 ^a^	9.3 ± 14.3 ^b^
**Numerical density of neuropeptides granules on cell borders (per linear surface)**	11.1 ± 3.2 ^a,b^	5.2 ± 2.4 ^a^	3.1 ± 1.7 ^b^

## Data Availability

The datasets used and analyzed in the current study are available from the corresponding author upon reasonable request. The data are not publicly available due to privacy considerations.

## Supplementary Materials

The following supporting information can be downloaded at: https://www.mdpi.com/article/10.3390/brainsci14050476/s1, Figure S1. Light microscopy of PC12 cells. Representative LM pictures of PC12 untreated (A), treated with rotenone for 24 and 48 h (B,C). Magnification: 20×.
