# Supplementary material for: Ultrastructural Changes of Neuroendocrine Pheochromocytoma Cell Line PC-12 Exposed In Vitro to Rotenone
Source: Brain Sci. 2024 May 8;14(5):476. doi: 10.3390/brainsci14050476 (PMC11119447; doi:10.3390/brainsci14050476)
Supplement: Supplementary file 1 [file brainsci-14-00476-s001.zip › brainsci-2985906-supplementary.pdf]

brain sciences

MDPI

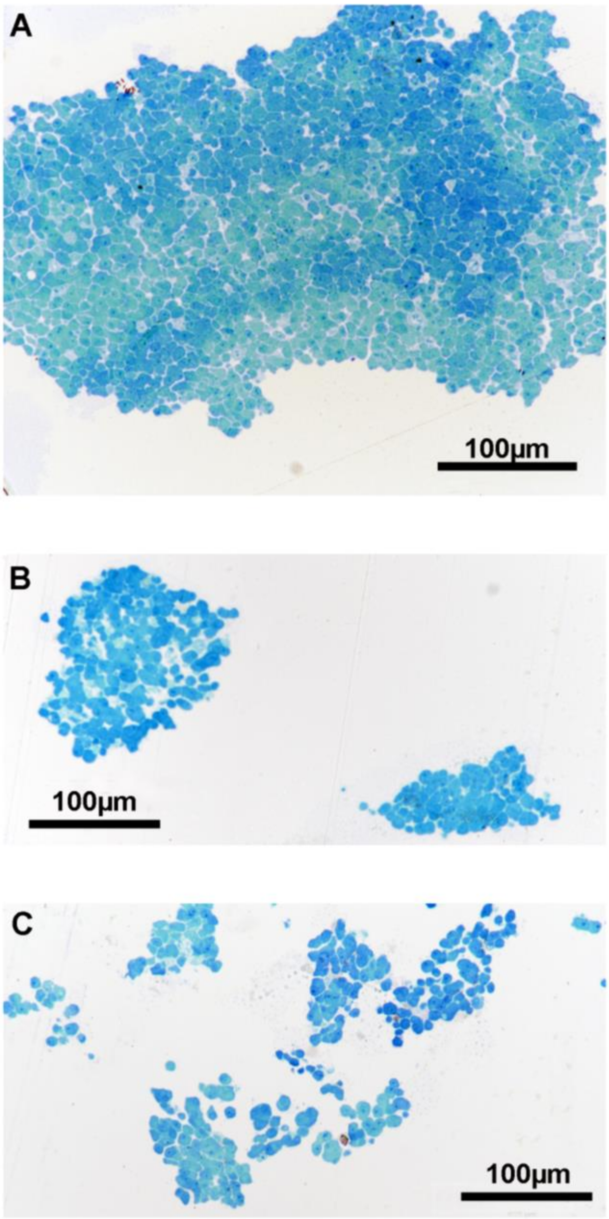

Supplementary Figure S1. Light microscopy of PC12 cells. Representative LM pictures of PC12 untreated (A), treated with rotenone 24 – and 48 hours (B-C). Magnification: 20x.
